# MM-ChIP enables integrative analysis of cross-platform and between-laboratory ChIP-chip or ChIP-seq data

**DOI:** 10.1186/gb-2011-12-2-r11

**Published:** 2011-02-01

**Authors:** Yiwen Chen, Clifford A Meyer, Tao Liu, Wei Li, Jun S Liu, Xiaole Shirley Liu

**Affiliations:** 1 Department of Biostatistics and Computational Biology, Dana-Farber Cancer Institute and Harvard School of Public Health, 44 Binney Street, Boston, MA 02115, USA; 2 Division of Biostatistics, Dan L Duncan Cancer Center, Department of Molecular and Cellular Biology, Baylor College of Medicine, One Baylor Plaza, Houston, TX 77030, USA; 3 Department of Bioinformatics, School of Life Science and Technology, Tongji University, Shanghai, 200092, PR China; 4 Department of Statistics, Harvard University, 1 Oxford Street, Cambridge, MA 02138, USA

## Abstract

The ChIP-chip and ChIP-seq techniques enable genome-wide mapping of *in vivo *protein-DNA interactions and chromatin states. The cross-platform and between-laboratory variation poses a challenge to the comparison and integration of results from different ChIP experiments. We describe a novel method, MM-ChIP, which integrates information from cross-platform and between-laboratory ChIP-chip or ChIP-seq datasets. It improves both the sensitivity and the specificity of detecting ChIP-enriched regions, and is a useful meta-analysis tool for driving discoveries from multiple data sources.

## Background

Chromatin immunoprecipitation (ChIP) followed by array hybridization (ChIP-chip) and ChIP followed by massively parallel sequencing (ChIP-seq) are two powerful techniques for profiling *in vivo *DNA-protein interactions [1,2] and histone marks on a genome-wide scale [3,4]. The genome-scale data generated by these two technologies provide information essential to our understanding of the transcriptional regulation underlying various cellular processes.

ChIP-chip/seq experiments are often performed on different technical platforms in different labs. Even ChIP-chip/seq data for the same protein under similar biological conditions can show significant variation between laboratories and across platforms due to differences in ChIP experimental protocols and platform designs [5]. Such variation can lead to platform- or lab-specific false positives/negatives, making it difficult to compare and integrate results from different ChIP experiments, despite the development of computational methods for analyzing ChIP data from individual sources separately [6-14].

To address this challenge, we have developed a new computational method and its companion software, named MM-ChIP (Model-based Meta-analysis of ChIP data), which enables the integrative analysis of ChIP-chip/seq data across platforms and between laboratories.

## Results

### Integrative analysis of ChIP-chip data

Currently, the most popular platforms for performing ChIP-chip experiments are high-density oligonucleotide tiling microarrays from Affymetrix, NimbleGen, and Agilent. These platforms differ greatly in probe lengths, tiling resolutions, and sample-labeling protocols, which results in platform-specific systematic bias (for example, probe-specific behavior and dye bias) and differences in noise features, detection sensitivity and dynamic range [5]. These differences make it difficult to effectively combine different datasets for detecting regions of enrichment.

**Figure 1:**
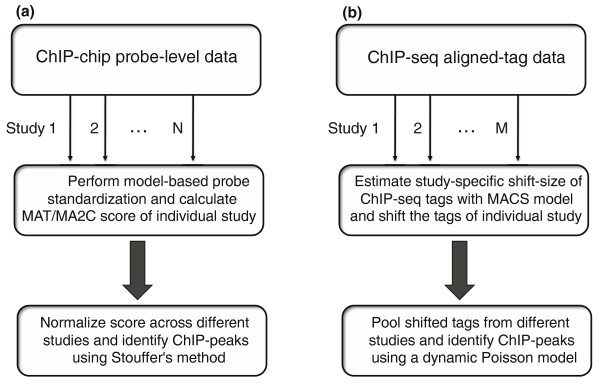
**The workflow of MM-ChIP**. Workflow illustrated for **(a) **ChIP-chip **(b) **and ChIP-seq data. MA2C, Model-based Analysis of 2-Color Arrays; MACS, Model-based Analysis of ChIP-Seq data; MAT, Model-based Analysis of Tiling-array.

To effectively take into account inter-platform differences and allow for the normalization of data from different sources, we designed a two-step process (Figure 1a). In the first step, raw probe-level data pooled from replicates are fitted to a platform-specific baseline probe model for each data source to remove the effect of probe sequence and genome copy number on probe intensity, a correction that has been shown to be important for increasing the signal-to-noise ratio [13,14]. A sliding window-based statistical score that summarizes the corrected probe intensity value within the window is then used to quantify ChIP signal enrichment at different genomic loci (Materials and methods).

**Figure 2:**
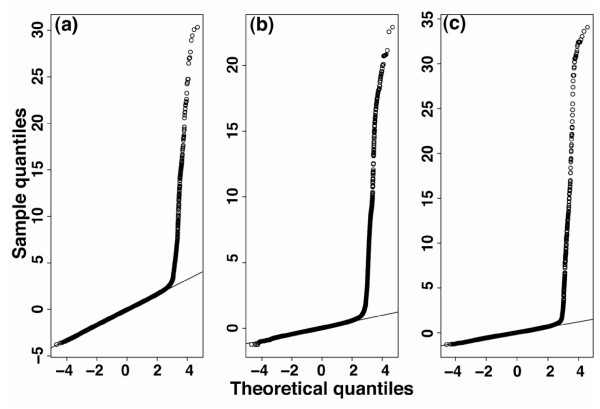
**Normal Q-Q plots of MAT/MA2C score distribution of three ChIP-chip datasets**. ChIP-chip datasets generated on **(a) **Affymetrix, **(b) **NimbleGen and **(c) **Agilent platforms are shown. MA2C, Model-based Analysis of 2-Color Arrays; MAT, Model-based Analysis of Tiling-array.

In the second step, the window-based scores are converted to a Z-score for each individual data source. The Z-scores corresponding to the same genomic loci across different data sources are summed to give a composite score and divided by the square root of the number of datasets, a calculation known as Stouffer's method [15]. Under the null hypothesis of no enrichment, this composite score is distributed as a standard normal distribution. The use of the Z-score for normalization and the choice of Stouffer's method were motivated by the observation that the distribution of window-based scores is approximately normal, with a heavy right tail irrespective of technical platform (Figure 2).

**Figure 3:**
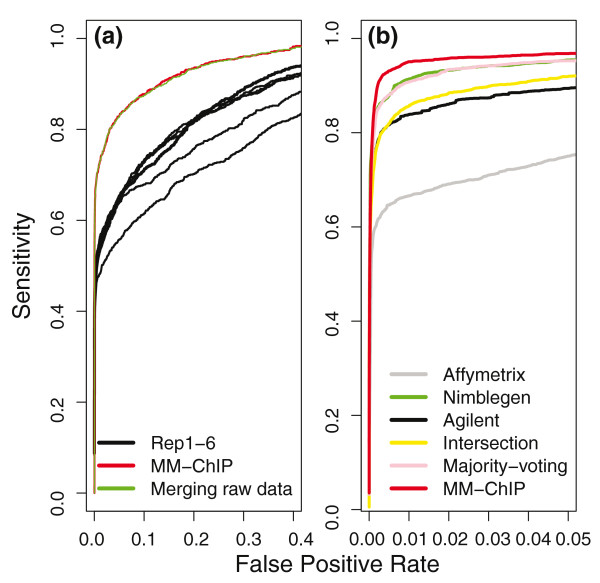
**An evaluation of the performance of MM-ChIP on ChIP-chip data is shown**. **(a) **ROC curves of the analyses performed using either individual replicates or all replicates from a single ChIP-chip dataset generated using an Affymetrix array are plotted. **(b) **ROC curves of analyses from individual datasets and all three datasets are plotted. The integrative analyses on all three datasets were performed using MM-ChIP (red), majority voting (pink) or the region intersection method (yellow).

To assess the performance of MM-ChIP on ChIP-chip data, we used three ChIP-chip datasets that were generated by three labs from the same ENCODE (ENCyclopedia Of DNA Elements) spike-in sample using different array platforms [5,16]. The spike-in samples contained 100 cloned genomic DNA sequences (average length 497 bp) mixed with human genomic DNA, and the genomic DNA without the spike-in served as the control. We first evaluated the performance of MM-ChIP on integrating replicate data from the same dataset (that is, from the same lab and platform). Because we knew which genomic regions were actually enriched in the spike-in sample, we were able to plot receiver operating characteristic (ROC) curves for the evaluation. We found that by integrating information from multiple replicates, MM-ChIP improved both the sensitivity and specificity of detecting known enriched regions compared with using individual replicates. Its performance matched that of pooling the raw data from replicates for enriched region detection (Figure 3a). With this confirmatory result, we extended our evaluation to the integrative analysis of cross-platform and between-laboratory datasets. We found that, similar to the results of integrating replicates from a single data source, integrating data from three platforms and labs using MM-ChIP improved both the sensitivity and specificity of detecting ChIP-enriched regions over using individual datasets (Figure 3b).

We further compared MM-ChIP with two alternative methods, majority voting and region intersection, on the same spike-in dataset. In the majority voting method, a region is considered to have significant enrichment in the integrative analysis if it is enriched in more than half of the individually analyzed datasets. In the region intersection method, which is commonly used to combine results from different ChIP experiments, a region is considered to have significant enrichment if it is enriched in all individually analyzed datasets. We found that MM-ChIP outperforms both methods (Figure 3b). Notably, the majority voting method performed similarly to the best individual analysis and better than the region intersection method (Figure 3b), indicating that the common practice of region intersection is not an optimal solution for integrative analysis.

**Figure 4:**
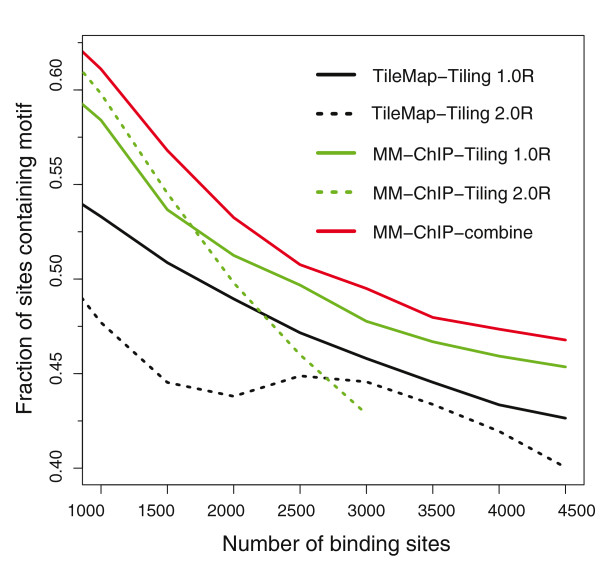
**An evaluation of the performance of MM-ChIP on two ER ChIP-chip datasets**. The fraction of ER binding sites that contain an ER motif is plotted as a function of the number of top-ranked binding sites for different cases using either MM-ChIP or TileMap.

After testing the performance of MM-ChIP on the spike-in datasets, we assessed its performance using two ChIP-chip datasets for the human estrogen receptor (ER). These two datasets were generated under the same biological conditions, but on two different array platforms: the Affymetrix Human Tiling 1.0R Array [17] and the Affymetrix Human Tiling 2.0R Array [18]. Because we did not know the enriched regions in these datasets *a priori*, we used enrichment of the ER binding motif to evaluate the quality of the inferred enriched regions. By mapping the occurrence of the ER binding motif within a 500-bp window surrounding the identified ChIP-chip peak summit, we found that the peaks identified by integrative analysis using MM-ChIP show consistently higher motif enrichment and thus improved peak-calling quality compared with those identified using individual datasets (Figure 4) with either MM-ChIP or the well-established tool TileMap. We chose TileMap for comparison because it has been shown to be among the best peak-calling tools for ChIP-chip data [19].

### Integrative analysis of ChIP-seq data

ChIP-seq [20-23] has become an important alternative technique to ChIP-chip with the emergence of next-generation sequencing platforms, such as the Illumina Genome Analyzer, Helicos HeliScope, and Applied Biosystems SOLiD. The Illumina Genome Analyzer is currently the most dominant platform, on which the vast majority of publicly available ChIP-seq datasets were generated. When sufficient sequencing depth is achieved, ChIP-seq has many advantages over ChIP-chip, including a much higher resolution, larger dynamic range, more complete genome coverage and presumably better signal-to-noise ratio.

**Figure 5:**
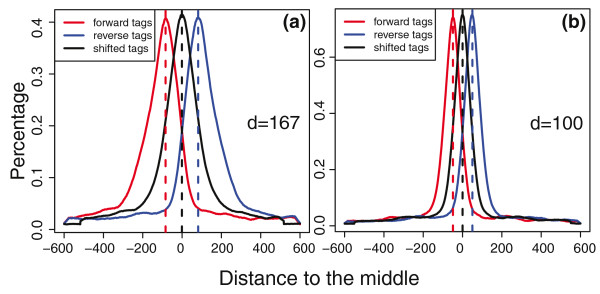
**MACS model of shift size for two CTCF ChIP-seq datasets**. Datasets were generated at **(a) **the Broad Institute and **(b) **the University of Texas at Austin through the ENCODE project.

Because ChIP-seq data have their own unique characteristics, we designed a different strategy for integrative peak detection compared with that for ChIP-chip (Figure 1b). ChIP-seq tags represent the ends of fragments in a ChIP-DNA library. The tag density around a true binding site generally shows a bimodal enrichment pattern, with Watson strand tags enriched upstream of binding and Crick strand tags enriched downstream [9,12]. To take into account this pattern and inter-study differences in ChIP-DNA library fragment size (Figure 5), MM-ChIP first models the characteristic fragment size of the sequenced ChIP-DNA library for each individual data source. The ChIP-seq tags are then shifted toward the 3' direction by a distance of half of the estimated fragment size to better represent the precise protein-DNA interaction sites.

Next, the model-shifted tags from different data sources are pooled for the ChIP and control samples independently. A sliding window is then used to score the significance of signal enrichment in the ChIP samples by comparing tags within the same window between the ChIP and control samples based on a dynamic Poisson model [12]. The use of this model was shown to reduce false positive detection because it can effectively capture local tag enrichment in the genome due to factors that are unrelated to the protein-DNA interaction of interest, such as local chromatin structure, copy number variation, and sequencing bias [12]. Because MM-ChIP only utilizes the 5' end positional information of each pooled tag for integrative analysis, it allows for the analysis of datasets that consist of tags with different read lengths, as long as the tags have been mapped to the same reference genome.

**Figure 6:**
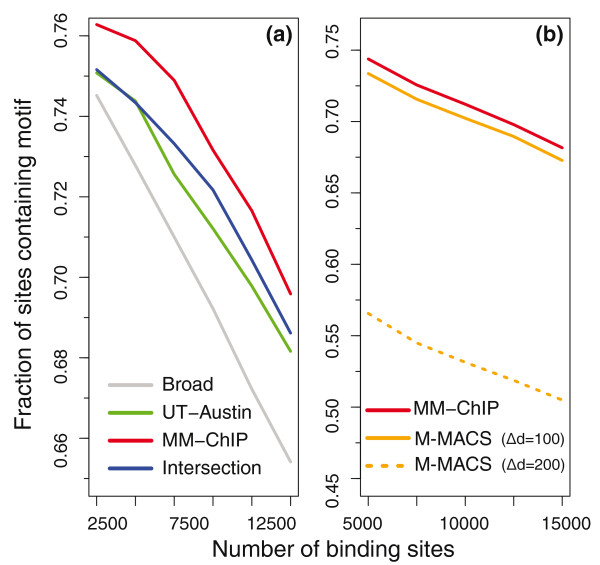
**An evaluation of the performance of MM-ChIP on ChIP-seq data**. **(a) **The fraction of CTCF binding sites containing a canonical CTCF binding motif is plotted as a function of the number of top-ranked binding sites for both the individual and combined datasets. The results of integrative analysis using the region intersection method are also shown. Binding sites were ranked in ascending order by *P*-value. Broad, Broad Institute; UT-Austin, University of Texas at Austin. **(b) **A comparison between MM-ChIP and the Merge MACS method on one real dataset and two synthetic datasets. The fraction of CTCF binding sites containing a canonical CTCF binding motif is plotted as a function of the number of top-ranked binding sites for different datasets when the inter-library size difference (Δd) is 0, 100 or 200. MACS, Model-based Analysis of ChIP-Seq data.

To assess the performance of MM-ChIP on ChIP-seq data, we used two recently released CCCTC-binding factor (CTCF) datasets from the ENCODE project [16]. Unlike with the spike-in ChIP-chip data, we did not know the true *in vivo *CTCF binding sites *a priori*. Therefore, we used enrichment of the canonical binding motif of CTCF to evaluate the performance of MM-ChIP for ChIP-seq peak detection. By mapping the occurrence of the CTCF binding motif within 50 bp of the identified ChIP-seq peak summit, we found that the peaks identified by integrative analysis using MM-ChIP showed consistently higher motif enrichment than those identified by using individual datasets, and MM-ChIP outperformed the region intersection method (Figure 6a). We also compared the performance of MM-ChIP with a workflow in which the first step of tag-shift was excluded, but the same procedures were performed in the second step. We found that exclusion of the tag-shift step in MM-ChIP significantly decreased its performance (Figure S1 in Additional file 1), which underscores the importance of modeling the fragment size of sequenced ChIP-DNA libraries.

In the two CTCF datasets described above, the fragment lengths did not differ considerably. However, in practice, different experimental protocols could yield distinct library sizes of 100 to 400 bp. We further compared the performance of MM-ChIP with an alternative method for the integrative analysis of datasets with varied inter-library size differences. The alternative method first merges the reads from different studies and then performs model building and peak detection using the MACS algorithm [12]. We chose this method for comparison because it is commonly used in practice. We found that the performance of MM-ChIP remains unchanged with varied inter-library size differences (Δd), whereas the performance of the alternative method deteriorates when Δd increases (Figure 6b). These results indicate that it is important to model the library size for individual studies separately before tag merging.

## Discussion

With the rapid increase in publicly available ChIP datasets, the development of computational methods for the integrative analysis of different ChIP datasets has become an emerging challenge. Two methods that are related to the current study have been developed recently. JAMIE (joint analysis of multiple ChIP-chip experiments) [24] is based on a hierarchical mixture model to capture correlations between datasets and allows for the joint analysis of multiple ChIP-chip datasets that are related to the same transcription factor. However, its current implementation only allows for the analysis of the datasets generated on the same array platform and does not support the integrative analysis of ChIP-seq datasets. In addition, JAMIE relies on a number of model assumptions about data and peak shapes that do not necessarily hold true for many ChIP-chip datasets. In contrast, MM-ChIP makes few assumptions about the statistical characteristics of ChIP-chip data and thus could be more robust.

Another method, hierarchical hidden Markov model (HHMM), is based on a hierarchical hidden Markov model and was developed specifically for the joint analysis of one ChIP-chip and one ChIP-seq dataset, using a Bayesian inference procedure [25]. However, HHMM does not effectively support the joint analysis of ChIP-chip datasets from different array platforms or ChIP-seq datasets with large inter-library heterogeneity. Moreover, its model complexity increases dramatically with the number of the datasets, whereas MM-ChIP is a deterministic approach with a computational complexity/time that scales linearly with the number of datasets. More importantly, the HHMM method uses the raw hybridization signal or tag count at each genomic location without effectively taking into account platform-specific biases, such as probe behavior and inter-study ChIP-DNA library heterogeneity, which could introduce significant systematic errors in the integrative analysis.

The current implementation of MM-ChIP weighs data from different sources equally in the integrative analysis. Given the heterogeneity in quality of different datasets, a more appropriate approach would be to weigh different data sources differently, according to some statistical measure of data quality. Stouffer's method provides a natural framework for treating data sources differently by using the weighted mean of the Z-scores. For example, if two datasets have comparable data qualities for individual replicates but different numbers of replicates, the weight can be proportional to the number of replicates in each dataset. However, how to generally incorporate information about the quality of individual data sources into an integrative analysis, especially for count data from ChIP-seq experiments, remains an important question.

An implicit assumption for using Stouffer's method in integrative analysis is that the Z-scores are independent among different datasets at non-enriched regions. This assumption does not necessarily hold because when datasets are generated from the same array platform and the probe effect is not completely removed by the Model-based Analysis of Tiling-array (MAT)/Model-based Analysis of 2-Color Arrays (MA2C) algorithm, any residual probe effect could cause an artificially enriched signal in the same genomic location across different datasets [26]. The aggregation of this signal could then lead to a false positive in the integrative analysis. When input control sample data are available, we expect that the residual probe effect has only a minor impact on the results of the analysis because it has a similar effect in non-enriched regions of the ChIP and input control samples, and its effects are cancelled out in the MAT/MA2C score. However, when there is no input sample, the residual probe effect could negatively affect the integrative analysis; thus, it is important to appropriately model and remove residual probe effects, as illustrated in a previous study [26].

Because of the lack of public ChIP-seq datasets for the same protein of interest under similar biological conditions from technical platforms other than Ilumina, our performance assessment of MM-ChIP was limited to Illumina datasets. Therefore, some caution needs to be taken when the method is applied to cross-platform datasets that are not generated on the Illumina platform. For ChIP-seq datasets across different sequencing platforms, different statistical models may be needed to account for inter-platform variations besides variation in inter-library size. Nonetheless, MM-ChIP is generally applicable to most publicly available ChIP-seq datasets because most of these datasets were generated on the Illumina platform.

MM-ChIP currently does not provide functionality for integrating data between array and sequencing platforms, but this will be an important direction to explore in the future. In addition to ChIP-chip/seq data, there are other types of genome-wide data, including microarray expression/RNA-seq data, which provide rich information for elucidating transcriptional regulatory networks. Most available integrative analysis methods, including MM-ChIP, are designed for a single data type. A challenge in the future will be developing methods for the integration of different data types from diverse sources.

## Conclusions

We have shown that integrating datasets from multiple sources using MM-ChIP improves both the sensitivity and the specificity of detecting ChIP-enriched regions. With the ever-increasing deposition of ChIP-chip/seq data into the public domain, MM-ChIP promises to become a powerful tool for biologists to make new discoveries that could not be achieved using a single data source (for example, finding weak but functional transcription factor binding sites and associated *cis*-regulatory modules from multiple sources of ChIP-chip/seq data).

## Materials and methods

### Dataset

Three ENCODE spike-in ChIP-chip datasets were used to assess the performance of MM-ChIP. The datasets were generated by Kevin Struhl's lab, Peggy Farnham's lab and Scott McCuine using Affymetrix, NimbleGen and Agilent tiling array platforms, respectively [5,16] [GEO:GSE10114]. To control for the effect of unbalanced replicate number in different studies, we chose similar numbers of replicates from each dataset (three replicates from the Affymetrix data, three replicates from the NimbleGen data and two replicates from the Agilent data) for integrative analysis and performance comparison. The two ER datasets from MCF7 cell lines were generated by two different groups using the Affymetrix Human Tiling 2.0R Array and the Affymetrix Human Tiling 1.0R Array [27,28]. For the dataset generated with the Tiling 2.0R array, two replicates each of ChIP and input data were used in our analysis. For the dataset generated with the Tiling 1.0R array, three replicates each of ChIP and input data were used in the analysis. The two CTCF ChIP-seq datasets from GM12878 cell lines were generated at the Broad Institute and at the University of Texas at Austin through the ENCODE project [16]. All ChIP-seq data from ENCODE and modENCODE (model organism ENCyclopedia Of DNA Elements) [29] projects were generated on the Illumina platform. To control for the effect of tag count difference, the same number of mapped tags (10,352,572) with unique genomic locations was selected from the ChIP and input samples from the two datasets.

### Integrative analysis of ChIP-chip data

#### Probe behavior model estimate and probe standardization for individual tiling array platforms

For the one-color Affymetrix platform, the MAT algorithm [13] was first used to fit the raw probe intensity to a baseline model to estimate the effect of probe sequence and genome copy number on intensity. The probe intensity value was then standardized to a t-value based on the fitted baseline model. Lastly, MAT computed a statistical score (MAT score) for individual sliding windows surrounding each tiled probe, and the difference in this score between the ChIP and input sample was used to quantify the relative ChIP signal enrichment [13]. If there was no input sample, the MAT score from the ChIP sample was used. For two-color platforms, including NimbleGen and Agilent, the MA2C algorithm [14] was first used to standardize the individual probe intensity value to a t-value by taking into account the effect of probe GC content on raw intensity (that is, modeling the GC-specific background hybridization intensities). Similar to MAT, MA2C then computed a statistical score (MA2C score) for a sliding window surrounding each tiled probe, and this score was used to quantify the relative ChIP signal enrichment [14].

#### Score normalization and integrative peak detection across different tiling arrays

To account for the difference in tiling resolution of different arrays, a linear interpolation was first performed to fill in the MAT/MA2C score (or MAT score difference between ChIP and input control sample) in matched genomic regions for all arrays. The interpolation was performed between two tiled probes only if they were spatially close to each other within a pre-defined distance based on the tiling resolution of the platform. For the spike-in datasets, the resolution was standardized to 7 bp, and the maximum distance between two tiled probes within which the interpolations were performed was 10 bp, 50 bp and 100 bp for Affymetrix, NimbleGen and Agilent, respectively. For the ER datasets, the resolution was standardized to be 35 bp, and the maximum distance between which the interpolations were performed was 50 bp. Because both the MAT and MA2C scores are approximately normally distributed, Z-scores were calculated based on the null distribution of MAT/MA2C scores to normalize the scores from different platforms. The estimation of the null distribution of MAT/MA2C scores was described in [13,14]. The sum of Z-score divided by the square root of the number of datasets, a calculation known as Stouffer's method [15], was used to quantify the ChIP signal enrichment. Under the null hypothesis of no enrichment, this score was distributed as a standard normal distribution, and a *P*-value was calculated accordingly [15]. The empirical false discovery rate (eFDR) of a peak list from ChIP-chip data is evaluated by MM-ChIP in a similar way to the MAT and MA2C algorithms: for a given Z-score cutoff value Z_0 _(Z_0 _> 0) that corresponds to the user-specified *P*-value, MM-ChIP finds all peaks with Z-scores greater than Z_0 _and all peaks with Z-scores less than -Z_0_. Then, the FDR is estimated as Number of negative Z-score peaks/Number of positive Z-score peaks. This FDR calculation is a slightly conservative estimate of the positive FDR proposed by Storey [30] (see Supplementary text in Additional file 1 for the detailed proof).

### Integrative analysis of ChIP-seq data

#### Model building and tag shifting for individual ChIP-seq datasets

The Model-based Analysis of ChIP-Seq data (MACS) algorithm [12] was first used to model the characteristic fragment size *d *of the ChIP-DNA library from each data source (Figure 4). MACS was then used to shift each ChIP-seq tag toward the 3' direction by a distance of half of the estimated fragment size (*d*/2) to better represent the precise protein-DNA interaction sites for that dataset.

#### Integrative peak detection using model-shifted tags from different ChIP-seq datasets

The model-shifted tags from each dataset were pooled together, and a sliding window-based approach similar to the one used in the MACS method [12] was used to detect candidate ChIP-enriched regions (peaks). The significance of a candidate peak was assessed based on a Poisson model with a dynamic lambda across the genome, which captures local biases in tag distribution [12]. The eFDR of a peak list from ChIP-seq data is evaluated by MM-ChIP in a similar way to the MACS algorithm. For each *P*-value cut-off, MM-ChIP uses the same parameters to find the number of peaks in a ChIP sample compared with input control sample and vice versa. The eFDR is defined as Number of input control peaks/Number of ChIP peaks.

### Motif enrichment analysis

The CTCF position-specific weight matrix was mapped onto the human genome using CisGenome [19] with a third-order Markov background model.

### Performance evaluation of integrative analysis of ChIP-seq with varied inter-library size differences

The performance of MM-ChIP and an alternative method that first merges the reads from different studies and then performs model building and peak detection using the MACS algorithm were evaluated on synthetic CTCF ChIP-seq datasets with varied inter-library size differences (Δd). To generate a series of synthetic datasets with varied Δd values, the University of Texas at Austin ChIP-seq tags (library size d = 100) were first equally divided into two groups by random tag selection. One group of tags was used as common library data (d = 100) for all datasets. The tags in the remaining group were shifted toward the 5' direction by various distances to constitute the variant library data. An integrative analysis was performed on each pair of common library and variant library data (Δd = 0, 100, 200) to evaluate the performance of both algorithms.

### Software availability

The companion software for MM-ChIP was written in Python and can be downloaded from the following link [31].

## Abbreviations

bp: base pair; ChIP: chromatin immunoprecipitation; CTCF: CCCTC-binding factor; eFDR: empirical false discovery rate; ENCODE: ENCyclopedia Of DNA Elements; ER: estrogen receptor; FDR: false discovery rate; HHMM: hierarchical hidden Markov model; JAMIE: joint analysis of multiple ChIP-chip experiments; MA2C: Model-based Analysis of 2-Color Arrays; MACS: Model-based Analysis of ChIP-Seq data; MAT: Model-based Analysis of Tiling-array; MM-ChIP: Model-based Meta-analysis of ChIP data; ROC: receiver operating characteristic.

## Authors' contributions

YC and XSL conceived the project and wrote the manuscript. YC designed and implemented the algorithms and wrote the software package. All authors participated in the discussions and contributed to the analysis of the intermediate results throughout the project.

## Supplementary Material

Additional file 1 **Supplementary Figure S1 and supporting text**. Additional file 1 contains Supplementary Figure S1 and supporting text that describes false discovery rate calculation for integrative analysis based on Stouffer's method. Click here for file
