# Supplementary material for: MM-ChIP enables integrative analysis of cross-platform and between-laboratory ChIP-chip or ChIP-seq data
Source: Genome Biol. 2011 Feb 1;12(2):R11. doi: 10.1186/gb-2011-12-2-r11 (PMC3188793; doi:10.1186/gb-2011-12-2-r11)
Supplement: Additional file 1 — Supplementary Figure S1 and supporting text. Additional file 1 contains Supplementary Figure S1 and supporting text that describes false discovery rate calculation for integrative analysis based on Stouffer's method. [file gb-2011-12-2-r11-S1.DOC]

**Chen *et al.* MM-ChIP enables integrative analysis of cross-platform and between-laboratory ChIP-chip or ChIP-seq data Supplementary figure and supporting text**

**Figure S1.** The CTCF motif enrichment in binding sites that were identified with or without tag-shift by MM-ChIP is compared. The fraction of the CTCF binding sites that contain canonical CTCF sequence motif is plotted as a function of the number of top ranked binding sites.


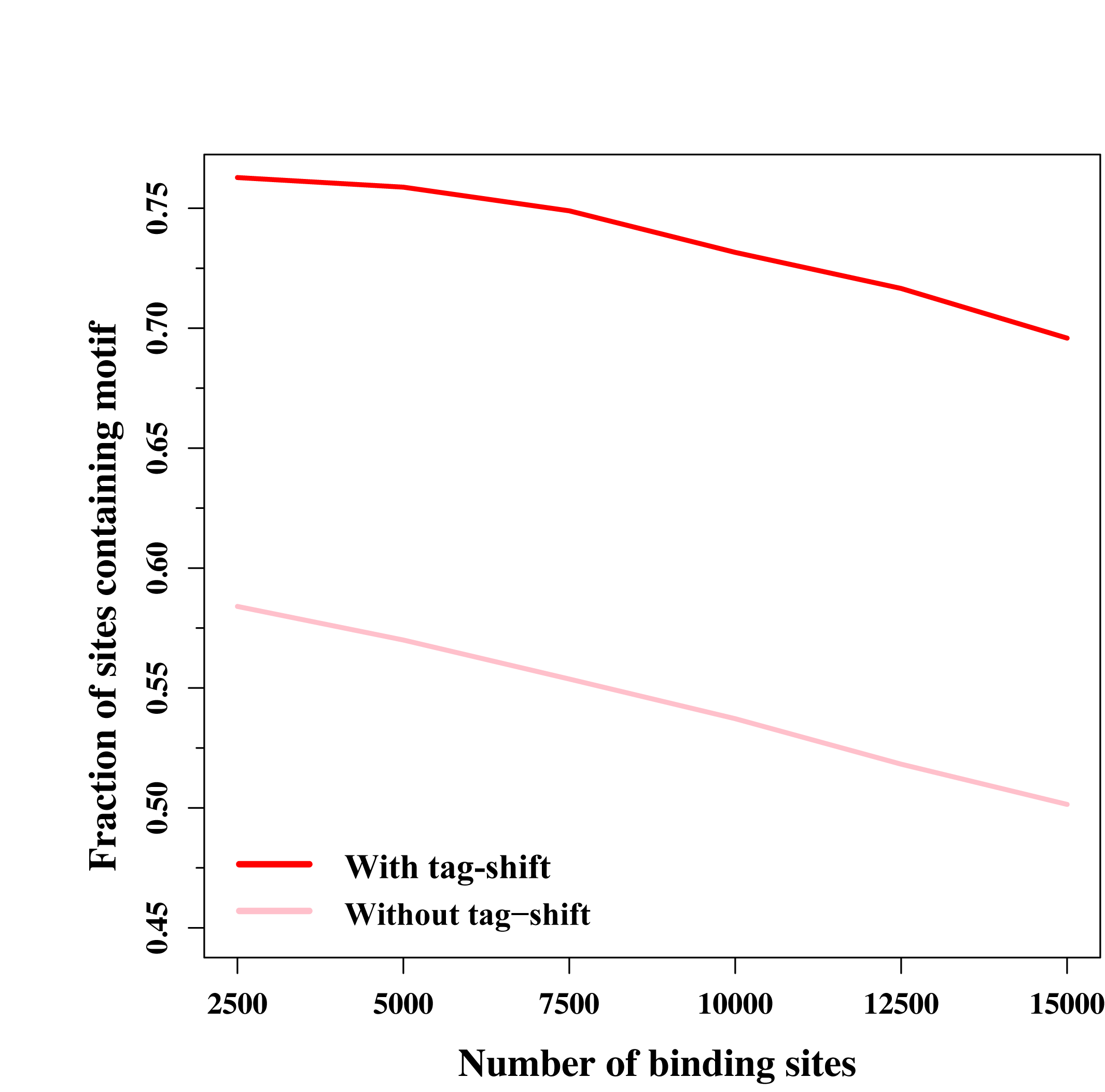


**Supporting text**

**FDR calculation for integrative analysis based on Stouffer’s method**

This FDR calculation is a slightly conservative estimate of the positive false discovery rate (pFDR) proposed by Storey[1]. The basic idea is that the # of false positive peaks in the FDR calculation can be approximately estimated as the # of negative peaks under several assumptions. The proof is as follows:

We denote by$I_{i}$ the indicator variable of the ith region ($I_{i}=1$, if the ith region is truly enriched in ChIP sample, otherwise, $I_{i}=0$) and $Z(x_{i},y_{i})$ the composite Z-score of the ith region where $x_{i}$,$y_{i}$ are corresponding MAT/MA2C scores in ChIP and input control samples. Based on the Bayesian interpretation of pFDR[29], it follows that


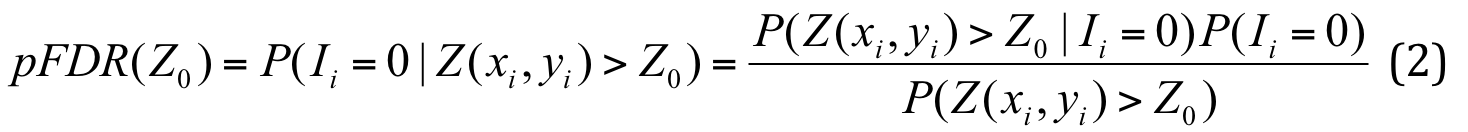


by applying Bayes’ rule.

Because $Z(x_{i},y_{i})$ follows the standard-normal distribution under the null model, it follows that

From equation (4), it follows that

We further made the assumptions that

Inequality (6a) is a reasonable assumption because the scores from ChIP-enriched regions are dominantly in the positive tail of the overall score distribution, whereas the scores from non-enriched regions have much more significant portions in the negative tail. Inequality (6b) is based on the fact the ChIP-enriched regions are only a small fraction of the whole genome. Thus it follows that

Because of the inequality (7), the 2^nd^ term on the right side of the inequality (5) is negligible compared with the first term and it follows that

Therefore our FDR calculation is a slightly conservative estimate of the pFDR from Storey[29].
